# Cardiovascular-kidney-metabolic comorbidities in colorectal cancer survivors: a nationwide cohort study on the role of metabolic syndrome

**DOI:** 10.3389/fendo.2025.1629675

**Published:** 2025-07-10

**Authors:** Xin Liu, Wenbo Zhao, Chang Zheng

**Affiliations:** ^1^ Department of Pathology, The First Affiliated Hospital of Wenzhou Medical University, Wenzhou, China; ^2^ Department of Clinical Epidemiology and Center of Evidence Based Medicine, The First Hospital of China Medical University, Shenyang, China

**Keywords:** colorectal cancer, cancer survivorship, metabolic syndrome, cardiovascular disease, CKM comorbidities

## Abstract

**Background:**

Colorectal cancer (CRC) survivors are at increased risk of cardiovascular-kidney-metabolic (CKM) complications. Although metabolic syndrome (MetS) is a recognized precursor of cardiovascular disease (CVD) in the general population, its prognostic relevance in CRC survivors has not been well established.

**Methods:**

We retrospectively analyzed 32,740 patients with a history of CRC who underwent health check-ups recorded in a national hospital-based database between 2005 and 2021. MetS was defined according to the Chinese Diabetes Society (2017) criteria, requiring central obesity (waist circumference ≥90 cm in men or ≥85 cm in women) and at least two of the following components: elevated blood pressure, hypertriglyceridemia, low high-density lipoprotein cholesterol, or elevated fasting glucose. Patients were grouped by MetS status at baseline and followed for composite CVD outcomes, including myocardial infarction, angina pectoris, stroke, and heart failure.

**Results:**

Among 32,740 CRC survivors (median age 58 years; 65.2% male), 4,970 (15.2%) met the criteria for MetS. Over a mean follow-up of 945 ± 770 days, 2,137 composite CVD events occurred. Incidence rates were significantly higher in the MetS group than the non-MetS group (368.2 vs. 200.8 per 10,000 person-years). In multivariable Cox models, MetS was independently associated with elevated CVD risk (HR = 1.36, 95% CI: 1.20–1.54). The association remained significant in male participants (HR = 1.43, 95% CI: 1.24–1.64), but not in females (HR = 1.09, 95% CI: 0.87–1.36). Subgroup analyses revealed consistent associations across strata of age, treatment status, and cancer type, with stronger effects observed in patients without obesity (P for interaction < 0.05). Among individual MetS components, elevated blood pressure and fasting glucose showed the strongest associations with CVD outcomes.

**Conclusions:**

Metabolic syndrome is a significant predictor of cardiovascular events among colorectal cancer survivors in China.

## 1 Introduction

Despite a downward trend in the average age of onset in high-income countries, colorectal cancer (CRC) remains the third most commonly diagnosed malignancy worldwide (1). Advances in screening, surgical techniques, and adjuvant therapies have markedly improved survival rates among CRC patients, leading to a rapidly expanding population of long-term cancer survivors (2, 3). As cancer-related mortality continues to decline, increasing clinical attention has turned toward non-cancer comorbidities that affect survivors’ long-term health. Among these, cardiovascular-kidney-metabolic (CKM) disorders—including heart failure, coronary artery disease, stroke, diabetes mellitus, and chronic kidney disease—have emerged as key contributors to overall morbidity and mortality in this population (4, 5).

Metabolic syndrome (MetS), characterized by central obesity, hypertension, dyslipidemia, and impaired glucose metabolism, is a well-established precursor of cardiovascular disease (CVD) in the general population (6). Beyond its cardiometabolic implications, MetS has also been closely linked to CRC incidence and prognosis (7). Meta-analytic evidence suggests that MetS significantly increases the risk of CRC development and cancer-specific mortality, with the effect particularly pronounced among men (8, 9).

However, prior studies assessing the relationship between MetS and CVD risk in cancer survivors have largely focused on breast cancer or mixed cancer populations (10, 11), limiting their generalizability to CRC. Notably, East Asian CRC survivors, who exhibit distinct metabolic and body composition profiles, remain underrepresented in existing research. It is also unclear whether MetS confers elevated cardiovascular risk among non-obese CRC patients—an often-overlooked subgroup in risk stratification. Moreover, the relative contributions of individual MetS components to cardiovascular outcomes in CRC survivors remain insufficiently characterized.

To address these gaps, we conducted a large-scale retrospective cohort study using a national hospital-based dataset in China to examine the association between metabolic syndrome and cardiovascular risk among CRC survivors. We further investigated whether this relationship was modified by obesity status, age, sex, and treatment history, and identified the individual metabolic components most predictive of adverse cardiovascular outcomes.

## 2 Materials and methods

### 2.1 Study population

**Figure 1:**
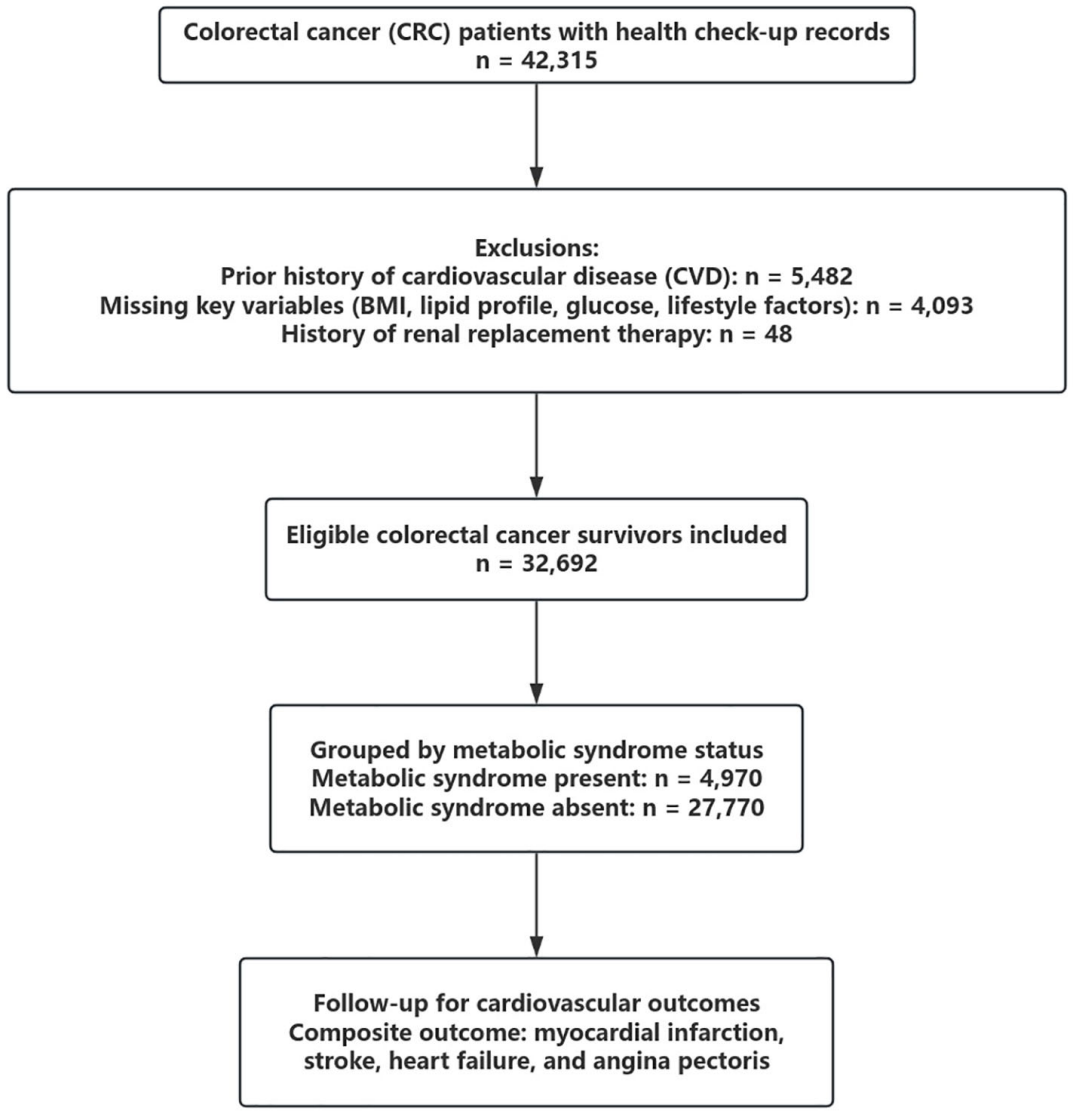
Flowchart of patient selection.

We retrospectively analyzed data from a large hospital-based health check-up and administrative claims database in China. A total of 42,315 patients with a diagnosis of colorectal cancer (CRC) who underwent at least one standardized health examination between January 2005 and December 2021 were initially screened. Individuals were excluded if they had a history of cardiovascular disease (n = 5,482), missing data on body mass index (BMI), lipid profile, fasting plasma glucose, smoking, alcohol use, or physical activity (n = 4,093), or a prior history of renal replacement therapy (n = 48). After exclusions, 32,740 eligible CRC survivors were included in the final analysis. A flowchart summarizing patient selection is shown in **Figure 1**.

This flowchart illustrates the inclusion and exclusion process for identifying eligible colorectal cancer (CRC) survivors. Of 42,315 patients with available health check-up records, individuals with a prior history of cardiovascular disease (n = 5,482), missing key variables (n = 4,093), or prior renal replacement therapy (n = 48) were excluded. A total of 32,740 CRC survivors were included and classified by metabolic syndrome (MetS) status at baseline.

### 2.2 Definition of metabolic syndrome

Metabolic syndrome (MetS) was defined according to the 2017 criteria issued by the Chinese Diabetes Society. A diagnosis of MetS required central obesity, defined as a waist circumference of ≥90 cm in men or ≥85 cm in women, along with at least two of the following components: elevated blood pressure (systolic ≥130 mmHg, diastolic ≥85 mmHg, or use of antihypertensive medication), hypertriglyceridemia (triglyceride level ≥1.7 mmol/L or current lipid-lowering therapy), reduced high-density lipoprotein cholesterol (HDL-C <1.04 mmol/L), or elevated fasting plasma glucose (≥6.1 mmol/L or use of antidiabetic treatment). All anthropometric and laboratory data were collected during standardized fasting health examinations. Lifestyle factors including smoking, alcohol consumption, and physical activity were assessed using structured self-reported questionnaires. Physical inactivity was defined as meeting either of two criteria: engaging in less than 30 minutes of moderate-intensity physical activity fewer than two times per week, or walking for less than one hour per day.

### 2.3 Outcome assessment

The primary outcome was defined as the first occurrence of a composite cardiovascular disease (CVD) event, including myocardial infarction, angina pectoris, stroke, or heart failure. Incident outcomes were identified through diagnostic codes from hospital claims data, based on the International Classification of Diseases, 10th Revision (ICD-10). Follow-up time was calculated from the date of the baseline health check-up to the date of the first cardiovascular event, death, or the end of the observation period in December 2021, whichever occurred first. Diagnostic criteria for metabolic syndrome are summarized in **Table 1**.

**Table 1:** Comparison of diagnostic criteria for metabolic syndrome: Chinese Diabetes Society (CDS 2017), International Diabetes Federation (IDF), and NCEP ATP III.

Criteria	Chinese Diabetes Society (CDS 2017)	International Diabetes Federation (IDF)	NCEP ATP III
Central Obesity	Waist ≥90 cm (men), ≥85 cm (women) *(Required)*	Required: Waist ≥90 cm (men), ≥80 cm (women, Asian-specific)	Waist ≥102 cm (men), ≥88 cm (women) *(Not required)*
Elevated Triglycerides	≥1.7 mmol/L (150 mg/dL)	≥1.7 mmol/L (150 mg/dL) or on treatment	≥1.7 mmol/L (150 mg/dL) or on treatment
Low HDL Cholesterol	<1.04 mmol/L (40 mg/dL)	<1.03 mmol/L (men), <1.29 mmol/L (women), or on treatment	<1.03 mmol/L (men), <1.29 mmol/L (women), or on treatment
Elevated Blood Pressure	≥130/85 mmHg or on antihypertensive medication	≥130/85 mmHg or on antihypertensive medication	≥130/85 mmHg or on antihypertensive medication
Elevated Fasting Glucose	FPG ≥6.1 mmol/L or 2hPG ≥7.8 mmol/L, or on antidiabetic treatment	FPG ≥5.6 mmol/L or on antidiabetic treatment	FPG ≥6.1 mmol/L or on antidiabetic treatment
Diagnosis Threshold	At least 3 of 5 components	Central obesity + any 2 other components	At least 3 of 5 components

This table summarizes the definitions of metabolic syndrome proposed by the Chinese Diabetes Society (2017 revision), the International Diabetes Federation (IDF), and the National Cholesterol Education Program Adult Treatment Panel III (NCEP ATP III). The CDS criteria use lower waist circumference cutoffs adapted to the Chinese population and define central obesity as a mandatory component. In contrast, the IDF also requires central obesity but applies a slightly different threshold for women. NCEP ATP III considers all five components equally and does not mandate central obesity. The selection of diagnostic criteria may influence the estimated prevalence of metabolic syndrome and its association with clinical outcomes.

### 2.4 Statistical analysis

Baseline characteristics were summarized using medians with interquartile ranges (IQRs) for continuous variables and frequencies with percentages for categorical variables. Between-group differences (MetS vs. non-MetS) were assessed using the Wilcoxon rank-sum test for continuous variables and the chi-square test for categorical variables. Kaplan–Meier methods were applied to estimate the cumulative incidence of cardiovascular disease (CVD) events, with differences compared using the log-rank test. Cox proportional hazards regression was used to estimate hazard ratios (HRs) and 95% confidence intervals (CIs) for the association between MetS and CVD. Three models were constructed: Model 1 was unadjusted; Model 2 adjusted for age and sex; and Model 3 further adjusted for body mass index (BMI), low-density lipoprotein cholesterol (LDL-C), smoking status, alcohol consumption, physical inactivity, cancer site, and a binary indicator of whether participants had received any form of cancer-related therapy within 6 months before the cardiovascular event or censoring. However, the dataset did not contain detailed information on treatment modality, dosage, or duration. Subgroup analyses were conducted by age (<50 vs. ≥50 years), obesity status (BMI <25 vs. ≥25 kg/m²), treatment exposure, and cancer site. Interaction terms were evaluated using multiplicative models, with P-values <0.10 considered suggestive of effect modification. Additionally, each component of MetS was analyzed separately to determine its independent association with cardiovascular outcomes. All statistical analyses were performed using Stata version 17.0 (StataCorp LLC, College Station, TX, USA), with two-sided P-values <0.05 considered statistically significant.

## 3 Results

### 3.1 Baseline characteristics

A total of 32,740 colorectal cancer (CRC) survivors were included in the final analysis, of whom 4,970 (15.2%) met the diagnostic criteria for metabolic syndrome (MetS). The median age of the study population was 58 years, and 65.2% were male. Compared with MetS-negative patients, those with MetS were older (61 vs. 57 years), had higher body mass index (27.4 vs. 22.5 kg/m²), waist circumference (95.5 vs. 81.2 cm), blood pressure (139/87 vs. 122/75 mmHg), fasting plasma glucose (6.7 vs. 5.4 mmol/L), and triglyceride levels (2.3 vs. 1.2 mmol/L), and lower high-density lipoprotein cholesterol (1.02 vs. 1.32 mmol/L). Additionally, patients with MetS were more likely to be male (74.7% vs. 63.3%) and reported higher rates of current smoking (18.5% vs. 13.9%), regular alcohol consumption (28.0% vs. 21.5%), and physical inactivity (58.2% vs. 45.0%). During the follow-up period, the proportion of composite cardiovascular disease (CVD) outcomes was significantly higher in the MetS group compared with the non-MetS group (12.3% vs. 5.5%) (**Table 2**).

**Table 2:** Baseline characteristics of colorectal cancer survivors stratified by metabolic syndrome status.

Variable	MetS (–) (n = 27770)	MetS (+) (n = 4970)	P-value
Sample size (n)	27770	4970	
Age, years (median [IQR])	57 (50–63)	61 (55–66)	<0.001
Sex, male (%)	17583 (63.3%)	3714 (74.7%)	<0.001
Body mass index, kg/m²	22.5 (20.6–24.8)	27.4 (25.5–30.1)	<0.001
Waist circumference, cm	81.2 (74.5–86.9)	95.5 (91.3–99.6)	<0.001
Systolic blood pressure, mmHg	122 (112–132)	139 (130–147)	<0.001
Diastolic blood pressure, mmHg	75 (68–83)	87 (80–95)	<0.001
Fasting plasma glucose, mmol/L	5.4 (4.9–5.9)	6.7 (6.0–7.8)	<0.001
Triglycerides, mmol/L	1.2 (0.9–1.6)	2.3 (1.6–3.0)	<0.001
High-density lipoprotein cholesterol, mmol/L	1.32 (1.14–1.50)	1.02 (0.89–1.17)	<0.001
Low-density lipoprotein cholesterol, mmol/L	3.1 (2.6–3.6)	3.4 (2.8–3.9)	0.015
Current smoker (%)	3860 (13.9%)	918 (18.5%)	<0.001
Alcohol consumption, regular (%)	5980 (21.5%)	1390 (28.0%)	<0.001
Physical inactivity (%)	12497 (45.0%)	2893 (58.2%)	<0.001
CVD outcome during follow-up (%)	1526 (5.5%)	611 (12.3%)	<0.001

### 3.2 Metabolic syndrome status

Metabolic syndrome (MetS) was defined according to the criteria of the Chinese Diabetes Society (2017 revision). Continuous variables are presented as median and interquartile range (IQR), and categorical variables are shown as frequency and percentage. P-values were calculated using the Wilcoxon rank-sum test for continuous variables and the chi-square test for categorical variables.

CVD outcome refers to the composite endpoint including myocardial infarction, angina pectoris, stroke, and heart failure, recorded during the follow-up period.

### 3.3 Cumulative incidence and overall cardiovascular risk

**Figure 2:**
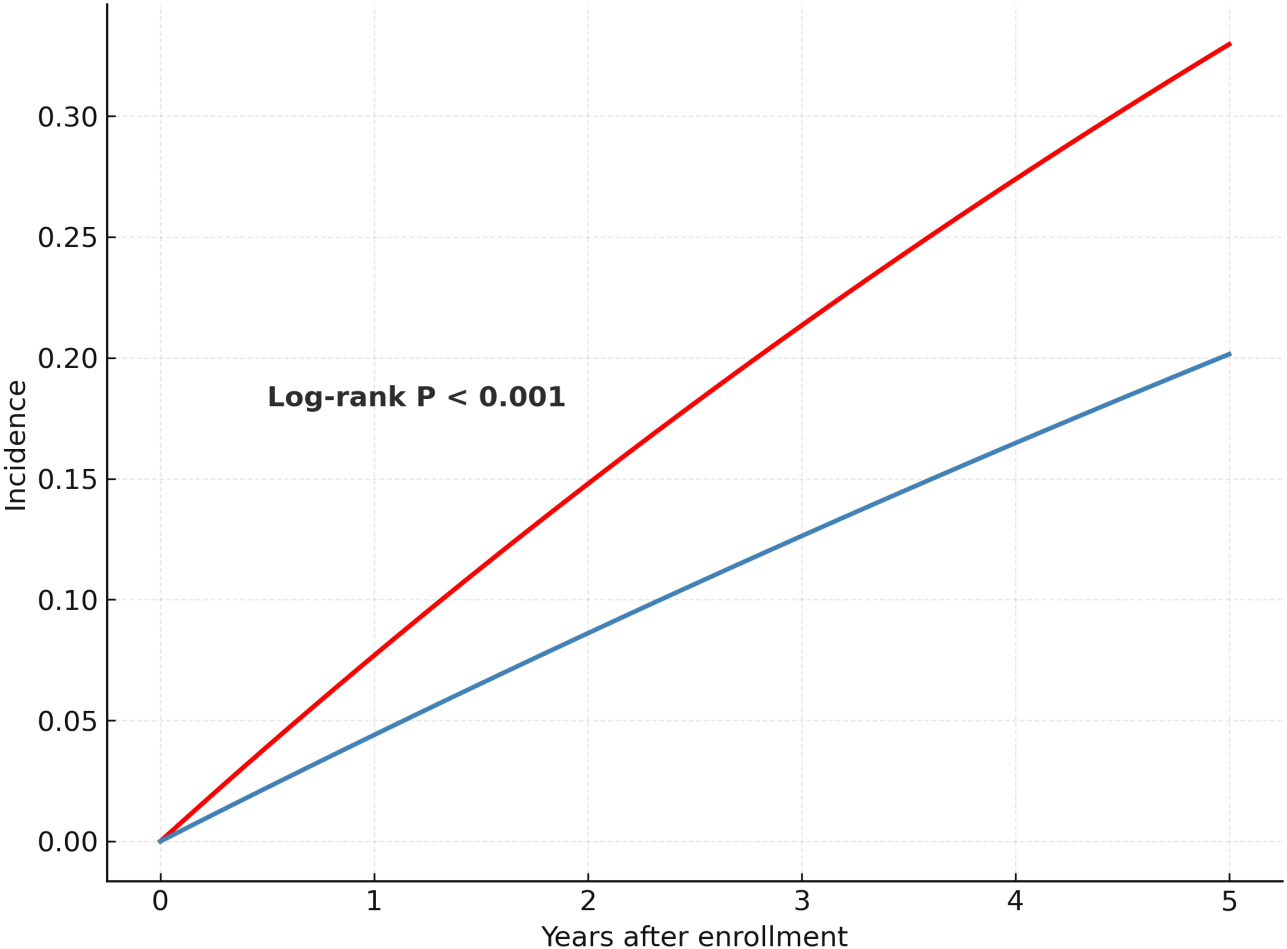
Kaplan–Meier curves for cumulative incidence of cardiovascular disease by metabolic syndrome status.

Over a mean follow-up duration of 945 ± 770 days, 2,137 composite CVD events were documented. Kaplan–Meier analysis showed a significantly higher cumulative incidence of cardiovascular events in patients with MetS compared with those without MetS (log-rank P < 0.001) (**Figure 2**). In Cox regression analyses, MetS was associated with increased CVD risk in all models. In the unadjusted model (Model 1), the hazard ratio (HR) was 1.83 (95% confidence interval [CI]: 1.66–2.01). After adjusting for age and sex (Model 2), the HR was 1.47 (95% CI: 1.32–1.62), and in the fully adjusted model (Model 3), MetS remained significantly associated with CVD (HR = 1.36, 95% CI: 1.20–1.54). In sex-stratified analysis, MetS was associated with increased CVD risk in men (HR = 1.43, 95% CI: 1.24–1.64), but not in women (HR = 1.09, 95% CI: 0.87–1.36) (**Table 3**).

**Table 3:** Cardiovascular events and hazard ratios according to metabolic syndrome status.

Group	Metabolic Syndrome	N	CVD Events (n)	Incidence Rate (per 10,000 py)	HR (95% CI) - Model 1	HR (95% CI) - Model 2	HR (95% CI) - Model 3
Overall	Absent	27770	1526	200.8 (193.2–208.7)	1.00 (reference)	1.00 (reference)	1.00 (reference)
	Present	4970	611	368.2 (336.9–402.3)	1.83 (1.66–2.01)	1.47 (1.32–1.62)	1.36 (1.20–1.54)
Men	Absent	17583	1074	240.6 (226.6–255.4)	1.00 (reference)	1.00 (reference)	1.00 (reference)
	Present	3714	484	382.8 (346.4–423.1)	1.59 (1.41–1.78)	1.48 (1.32–1.66)	1.43 (1.24–1.64)
Women	Absent	10187	452	179.8 (171.0–189.1)	1.00 (reference)	1.00 (reference)	1.00 (reference)
	Present	1256	127	323.0 (266.8–391.1)	1.79 (1.47–2.18)	1.43 (1.17–1.74)	1.09 (0.87–1.36)

This table presents the incidence rates and hazard ratios (HRs) for composite cardiovascular disease (CVD) outcomes among colorectal cancer survivors stratified by the presence of metabolic syndrome (MetS). Incidence rates are expressed per 10,000 person-years and were calculated based on observed events during follow-up.

Hazard ratios and 95% confidence intervals (CIs) were estimated using Cox proportional hazards models:

Model 1: Unadjusted.

Model 2: Adjusted for age and sex.

Model 3: Fully adjusted for age, sex, body mass index, low-density lipoprotein cholesterol, current smoking status, alcohol consumption, physical inactivity, and receipt of cancer treatment within 6 months prior to CVD event or censoring.

In subgroup analyses by sex, sex was removed from the covariates. The reference group consisted of patients without metabolic syndrome.

The figure shows the cumulative incidence of composite cardiovascular events (including myocardial infarction, stroke, heart failure, and angina pectoris) among colorectal cancer survivors, stratified by the presence or absence of metabolic syndrome (MetS). Patients with MetS had a significantly higher risk of cardiovascular events than those without MetS (log-rank P < 0.001).

### 3.4 Subgroup analyses

**Figure 3:**
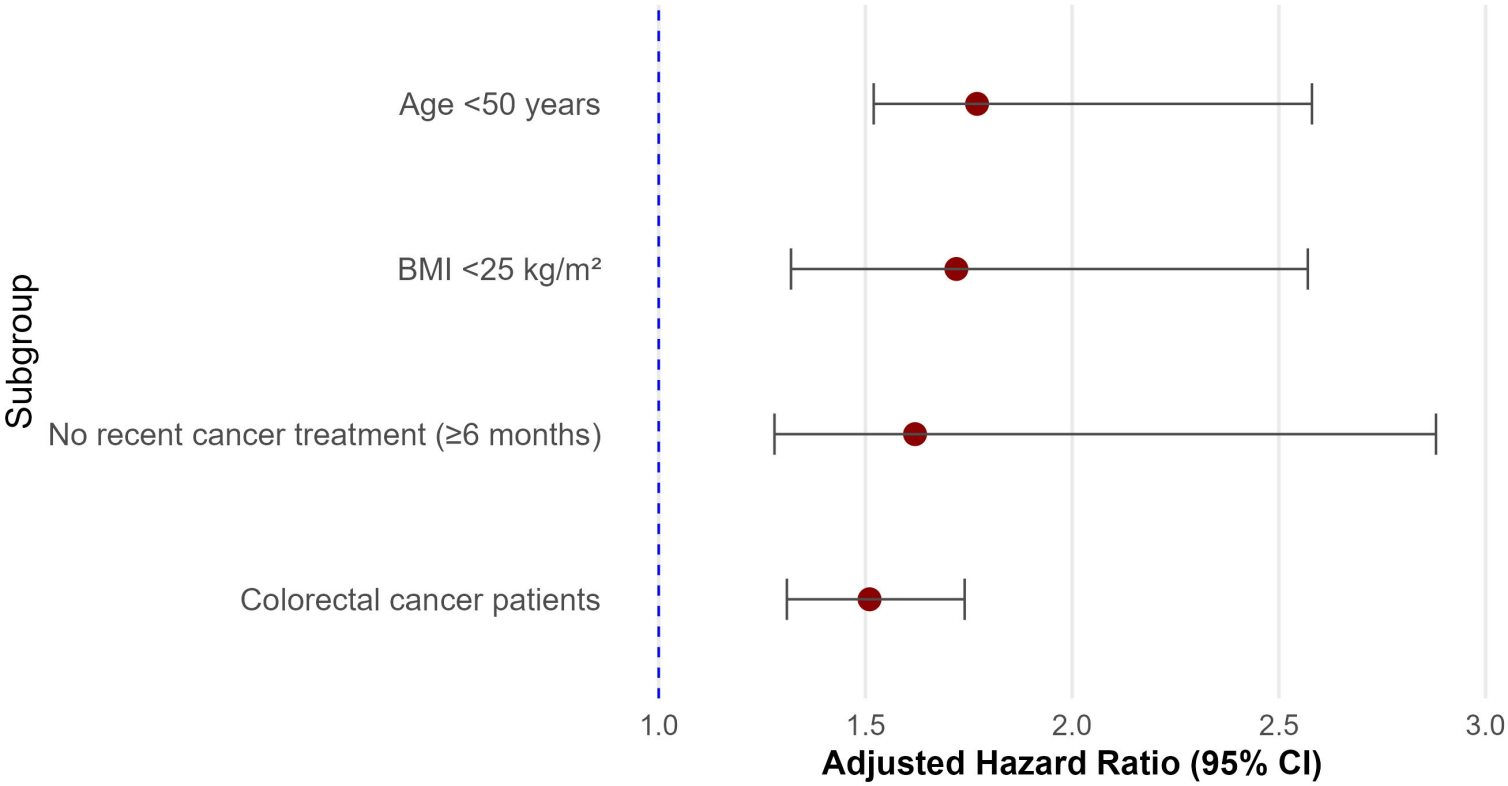
Subgroup analyses of the association between metabolic syndrome and cardiovascular disease (CVD) among colorectal cancer survivors.

Subgroup analyses demonstrated that the association between MetS and CVD was broadly consistent across key patient subgroups. The risk was more pronounced in participants aged <50 years (HR = 1.77, 95% CI: 1.52–2.58) and in those with BMI <25 kg/m² (HR = 1.72, 95% CI: 1.32–2.57). Participants who had not received recent cancer treatment within 6 months also exhibited stronger associations (HR = 1.62, 95% CI: 1.28–2.88) compared to those who had undergone treatment more recently. In cancer site–specific analysis, the association remained significant among colorectal cancer patients (HR = 1.51, 95% CI: 1.31–1.74). Detailed subgroup estimates, confidence intervals, and interaction values are presented in **Figure 3**.

This forest plot displays the adjusted hazard ratios (HRs) and 95% confidence intervals (CIs) for the association between metabolic syndrome (MetS) and CVD across predefined subgroups. Subgroups include age (<50 vs. ≥50 years), obesity status (based on BMI <25 kg/m²), recent cancer treatment (within 6 months), and internal stratifications within the colorectal cancer (CRC) cohort. Note that all participants were CRC survivors; subgroup comparisons reflect within-cohort stratifications. HRs were adjusted for age, sex, body mass index, LDL-C, smoking, alcohol use, physical inactivity, and treatment status. Incidence rates are expressed per 10,000 person-years.

Note: Subgroup sample sizes are not mutually exclusive and may overlap across stratifications; therefore, cumulative subgroup counts may exceed the total study population (N = 32,740).

CRC subsites were stratified as colon cancer and rectal cancer based on tumor location at diagnosis.

### 3.5 Cardiovascular disease

This forest plot displays the hazard ratios (HRs) and 95% confidence intervals (CIs) for the association between metabolic syndrome and cardiovascular disease across predefined subgroups. The association was stronger in younger patients (<50 years), non-obese individuals (BMI <25 kg/m²), and those without recent cancer treatment. Significant interactions were observed for age, obesity, and treatment status (*P* for interaction < 0.05).

### 3.6 Associations of individual MetS components with CVD

When individual components of the MetS criteria were analyzed, elevated blood pressure was the strongest predictor of incident CVD (HR = 1.39, 95% CI: 1.25–1.54), followed by elevated fasting glucose (HR = 1.22, 95% CI: 1.09–1.37). High waist circumference also demonstrated a modest but statistically significant association (HR = 1.18, 95% CI: 1.06–1.31). In contrast, neither elevated triglycerides (HR = 1.03, 95% CI: 0.94–1.12) nor reduced HDL-C (HR = 1.07, 95% CI: 0.93–1.17) were significantly associated with increased CVD risk. These findings were consistent in sex-stratified analyses, with elevated blood pressure and fasting glucose remaining significant predictors in both men and women (**Table 4**).

**Table 4:** Association of each component of the criteria for metabolic syndrome with incident cardiovascular disease in colorectal cancer survivors.

Component	Overall HR (95% CI)	Men HR (95% CI)	Women HR (95% CI)
High waist circumference	1.18 (1.06–1.31)	1.27 (1.11–1.45)	1.03 (0.86–1.24)
High blood pressure	1.39 (1.25–1.54)	1.52 (1.34–1.71)	1.23 (1.05–1.44)
Elevated TG	1.03 (0.94–1.12)	1.01 (0.92–1.11)	1.05 (0.90–1.21)
Reduced HDL-C	1.07 (0.93–1.17)	1.06 (0.91–1.23)	1.10 (0.93–1.29)
High blood glucose	1.22 (1.09–1.37)	1.20 (1.04–1.39)	1.25 (1.01–1.55)

Hazard ratios (HRs) and 95% confidence intervals (CIs) were estimated for individual components of metabolic syndrome, including high waist circumference, elevated blood pressure, elevated triglycerides, reduced HDL-C, and elevated fasting glucose. All models were adjusted for age, body mass index, low-density lipoprotein cholesterol, cigarette smoking, alcohol consumption, physical inactivity, cancer site, and recent cancer treatment (within 6 months). For sex-stratified analyses, sex was excluded from the covariates.

## 4 Discussion

In this large cohort of Chinese colorectal cancer (CRC) survivors, we found that metabolic syndrome (MetS), defined by national criteria, was independently associated with an increased risk of cardiovascular disease (CVD). This association remained robust after adjustment for demographic, behavioral, and clinical variables, and was especially pronounced in men, younger individuals, and those with normal BMI. Among MetS components, elevated blood pressure and fasting glucose were the strongest predictors of CVD events, whereas dyslipidemia showed no significant association.

These findings are consistent with previous evidence linking MetS to CVD in general populations (12–14) and extend the relevance of this association to CRC survivorship. Most prior studies have focused on breast cancer cohorts (15–17) or mixed cancer populations (16, 18–20), thereby limiting their applicability to CRC survivors. Although studies have assessed MetS severity and health impacts in older Chinese adults (21–23), few longitudinal investigations have specifically addressed CVD outcomes in this patient group.

An important observation in our study was that the MetS-associated increase in CVD risk was more evident in individuals with normal BMI, supporting the concept of the “metabolically obese normal-weight” (MONW) phenotype (24). This underscores the limitations of BMI-based risk stratification (25) and highlights the clinical value of assessing visceral adiposity and insulin resistance even in non-obese populations (26).

Although our dataset did not contain detailed information on cancer treatment modalities, prior research has shown that fluoropyrimidine-based chemotherapy agents such as 5-fluorouracil and capecitabine may impair endothelial function and induce hyperglycemia (27), while oxaliplatin and irinotecan are associated with dyslipidemia and insulin resistance (28). Pelvic radiotherapy has also been linked to chronic vascular inflammation and increased long-term CVD risk (29). These unmeasured treatment effects may act as potential confounders or mediators of the observed associations. In our models, we included a binary variable indicating whether participants had received any cancer-related therapy (surgery, chemotherapy, or radiotherapy) within 6 months prior to the cardiovascular event or censoring, in order to partially adjust for treatment exposure. Moreover, the observed sex-specific difference—with a significant association between MetS and CVD observed in men but not in women—might reflect several plausible biological and epidemiological reasons. First, estrogen may exert protective cardiovascular effects among females, particularly in premenopausal and early postmenopausal stages, mitigating the cardiovascular impact of metabolic abnormalities. Second, the smaller sample size and fewer cardiovascular events among females with MetS could have limited statistical power, reducing the likelihood of detecting significant associations. Additionally, differences in behavioral factors such as healthcare utilization, medication adherence, and lifestyle interventions between sexes might further influence cardiovascular risk outcomes. Future studies specifically addressing hormonal status and detailed lifestyle variables in women could help elucidate this observed discrepancy.

From a clinical standpoint, our findings support routine screening for MetS components among CRC survivors, regardless of age or BMI. Priority should be given to blood pressure and glucose control, which emerged as the most critical targets. Although the Chinese Diabetes Society criteria for MetS, utilized in this study, specifically use lower waist circumference thresholds (≥90 cm in men and ≥85 cm in women) tailored to Asian populations compared to international standards such as the ATP III (≥102 cm for men, ≥88 cm for women) and IDF (ethnicity-specific, generally ≥90 cm for Asian men and ≥80 cm for Asian women), the core components (central obesity, elevated blood pressure, hyperglycemia, and dyslipidemia) remain consistent across definitions (30, 31). Therefore, while absolute risk estimates might vary slightly when international criteria are applied, the overall observed associations between MetS and CVD outcomes are expected to remain similar and generalizable, especially to Asian and other comparable global populations.

Several limitations should be acknowledged. First, residual confounding from unmeasured factors—such as dietary habits, chronic inflammation, or genetic predisposition—cannot be excluded. Second, the observational design precludes causal inference. Third, since our data were obtained from a hospital-based health examination and administrative claims database, the study population may be more health-conscious or have better healthcare access than the broader CRC survivor population. This may introduce selection bias and limit generalizability. Fourth, although we adjusted for recent cancer treatment using a binary variable, we lacked detailed information on specific cancer therapy types, treatment intensity, duration, dietary patterns, and medication use. These additional unmeasured lifestyle and clinical factors could potentially confound or mediate the observed associations. Future studies incorporating these detailed confounders could enhance the robustness and interpretability of our findings. In addition, the average follow-up period of 945 days, with a large standard deviation of 770 days, suggests variability in observation time and may limit the assessment of long-term CVD outcomes. Future research should aim to integrate cancer treatment profiles, inflammatory biomarkers, and genetic data to enhance cardiovascular risk prediction and inform personalized survivorship care strategies.

## 5 Conclusion

This nationwide cohort study of colorectal cancer (CRC) survivors in China demonstrates that metabolic syndrome (MetS), as defined by national criteria, is independently associated with an increased risk of cardiovascular disease (CVD). The excess risk was particularly evident among men, younger individuals, and those with normal body mass index, suggesting the need to assess metabolic health beyond traditional obesity measures. Among individual MetS components, elevated blood pressure and fasting glucose emerged as the most powerful predictors of cardiovascular outcomes.

## Data Availability

The original contributions presented in the study are included in the article/supplementary Material. Further inquiries can be directed to the corresponding author.
